# Sublethal insecticide exposure of larvae affects the blood-feeding behaviour of adult mosquitoes

**DOI:** 10.1186/s13071-025-06815-x

**Published:** 2025-05-24

**Authors:** Tiago G. Zeferino, Gwendoline Acerbi, Jacob C. Koella

**Affiliations:** 1 Institute of Biology, University of Neuchâtel, Rue Emile-Argand 11, 2000 Neuchâtel, Switzerland; 2 Department of Evolutionary and Integrative Ecology, Leibniz Institute of Freshwater Ecology and Inland Fisheries (IGB), Berlin, Germany; 3 Institut Für Biologie, Freie Universität Berlin, Berlin, Germany

**Keywords:** Insecticide exposure, Mosquito behaviour, Blood-feeding, *Anopheles gambiae*, Vector control

## Abstract

**Background:**

Due to their widespread use for controlling disease vectors and agricultural pests, insecticides have become ubiquitous in the environment, including in water bodies harbouring mosquito larvae. As a result, these larvae are continuously exposed to sublethal doses. Since this has long-lasting effects on the mosquitoes’ physiology and life-history, we expected that it may also affect behaviours that underlie the mosquitoes’ population dynamics and disease epidemiology, such as egg-laying preference, blood-feeding motivation and host-seeking behaviour.

**Methods:**

Using an insecticide-sensitive and a resistant strain of *Anopheles gambiae*, an important malaria vector, we evaluated the effects of sublethal exposure to permethrin throughout larval development on the resistance to the insecticide in adults, on host-seeking behaviour, on the motivation to blood-feed and on egg-laying behaviour.

**Results:**

Exposure to sublethal doses of insecticide did not affect knock-down or mortality rates. However, it decreased the avoidance of permethrin-treated nets, and it increased the motivation of females to seek blood meals through permethrin-treated nets, regardless of their sensitivity to the insecticide. It also increased the blood-meal size in particular of the sensitive mosquitoes. Finally, exposed females were more likely than unexposed ones to lay their eggs into several sites.

**Conclusions:**

Sublethal insecticide exposure during larval development changes several aspects of the behaviour of mosquitoes in ways that could enhance disease transmission and may thus have significant epidemiological implications.

**Graphical Abstract:**

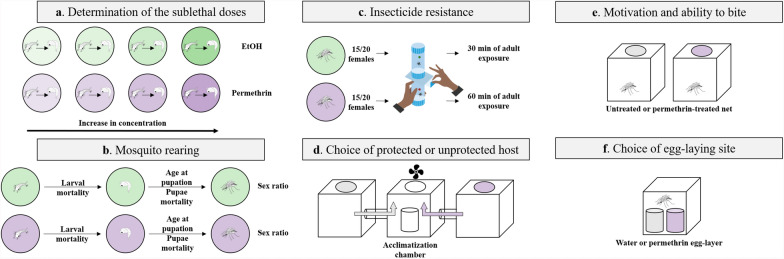

**Supplementary Information:**

The online version contains supplementary material available at 10.1186/s13071-025-06815-x.

## Background

Mosquitoes and other vectors of infectious diseases are often exposed to sublethal doses of insecticides. One reason is that these chemicals are extensively used to control mosquito-borne diseases such as malaria, in particular in combination with bed-nets [1, 2]. Therefore, in searching for a host, mosquitoes will touch a bed-net and can be irritated by the insecticide and fly away before the contact is long enough to kill them. Another reason is that some of the insecticides that are used in agriculture often end up in surface or ground water [3, 4], through various pathways such as atmospheric deposition, soil leaching [5] and volatilization or degradation [6], where they can affect many animals, including aquatic arthropods such as mosquito larvae.

Even if their concentration is too low to kill the larvae, these insecticides impact the adults by influencing their life-history traits [6–9], their behaviour [10–12] and their competence to transmit, for example, arboviruses [13, 14] and malaria parasites [15–17]. One possible mechanism is that insecticides disrupt key physiological pathways [18]. Insecticides, for example, impair the olfactory systems of mosquitoes [19, 20], thus impeding their responses to attractants involved in host-seeking and oviposition [11, 21–23]. Some effects of exposure to a low dose of insecticide can carry over to the next generation. In Colorado potato beetles, the offspring of mothers exposed to a low dose of a pyrethroid were larger as larvae and pupae than those of unexposed individuals [24]. By contrast, in cabbage aphids, exposure to a low dose of spirotetramat decreased body size and longevity in the offspring [25].

Despite this body of knowledge, little is known about how the exposure to a sublethal dose affects the later response of mosquitoes to insecticides. One exception is an experiment suggesting that repeated exposure to permethrin in adults does not change sensitivity to a subsequent exposure [26].

Considering that the stress of exposure affects the response to insecticides, it would not be too surprising for other stressors to do the same. Malaria-infected mosquitoes, for example, are less repelled by insecticide-treated nets, suggesting that they could also be less killed [27]. Larval crowding [28] or undernutrition [29] both also increase the phenotypic sensitivity of mosquitoes to insecticides.

In several experiments, we investigated how sublethal larval exposure to permethrin influences adult mosquitoes’ responses to insecticide in both an insecticide-resistant and a sensitive strain of *Anopheles gambiae*. Permethrin residues are often detected in surface waters, with concentrations ranging from 0.003 µg/L to 0.80 µg/L (median: 0.04 µg/L; 25th percentile: 0.01 µg/L; 75th percentile: 0.31 µg/L) as highlighted by an extensive review [3, 4]. Here, after assessing the sublethal dose for each strain under our laboratory conditions, we evaluated resistance by measuring knock-down rates and mortality using the World Health Organization (WHO) tube test. Additionally, we investigated the mosquitoes’ preference for a host protected by an insecticide-treated or untreated net, their motivation and ability to bite through a permethrin-treated net and their choice of insecticide-laced or untreated water as an oviposition site.

## Methods

### Experimental system

We used two strains of the mosquito *An. gambiae* (s.s) – Kisumu, a permethrin-sensitive strain [30], and reduced susceptibility to permethrin (RSP), a permethrin-resistant strain [31, 32] – to compare the effects of a prolonged larval exposure to a sublethal dose of the insecticide permethrin on insecticide resistance and various behaviours of adult mosquitoes. The different traits measured were considered in separate experiments that used the same protocol to rear larvae and the same concentration of permethrin (as described below). The resistance of RSP is a product of a *kdr* sodium channel mutation (L1014S), increased cytochrome P450 activity and elevated beta-esterase activity. L4 larvae are exposed to 0.5 mg/L of permethrin for 24 h every three generations to maintain permethrin resistance. We maintained the two strains in three cages, where generations overlapped with a 1-week age difference, at a density of about 600 individuals per cage and standard lab conditions (26 ± 1 °C; 70 ± 5% relative humidity; 12 h light/dark) for several years before the experiments.

### Determination of the sublethal doses

Since permethrin has a high absorption in plastic material and a low solubility in water [33], all experiments were performed with glass containers. The pyrethroid was dissolved in ethanol; so as to control for a potential negative effect of the ethanol, our controls contained the same concentration of ethanol as their respective permethrin treatments. An initial solution was prepared using solid permethrin (PESTANAL®, analytical standard, CAS No. 52645-53-1; Sigma-Aldrich, Inc., St. Louis, Missouri) dissolved in absolute ethanol at a concentration of 1 mg/L. This solution was used to form the diluted solutions at the concentrations described below.

**Fig. 1:**
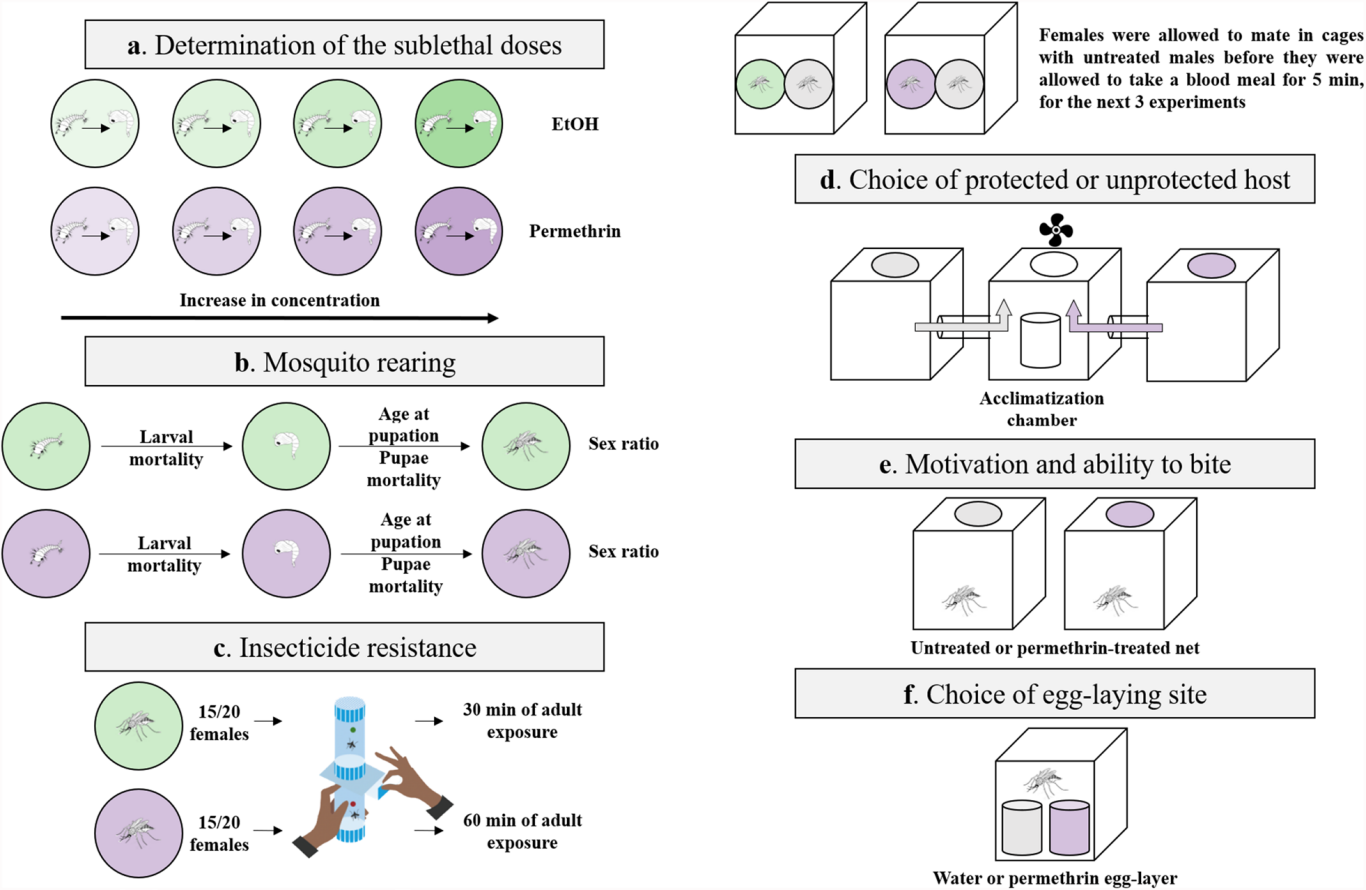
**Experimental design.** Illustration of the experiments in this study. **a** Determination of the sublethal doses. **b** Mosquito rearing. **c** Insecticide resistance. **d** Choice of protected or unprotected host, adapted from [27]. **e** Motivation and ability to bite through a permethrin-treated net. **f** Choice of egg-laying site

Larvae were exposed to either ethanol or ethanol supplemented with permethrin throughout their aquatic stage (Fig. 1a). The sublethal dose was defined as the highest tested concentration at which, during a preliminary experiment, the proportion of individuals that died as larvae did not differ significantly from the control solution under our laboratory conditions. For Kisumu, on the basis of the sublethal dose previously reported [17], we tested three concentrations (0.05, 0.15, and 0.45 µg/L) and found that 0.05 µg/L was the sublethal dose. For RSP, we tested 15 concentrations from 0.05 µg/L (the sublethal dose of Kisumu) to 100 µg/L and found that 0.75 µg/L was the sublethal dose. We used these doses in the experiments. For details on the concentrations, see Supplementary Fig. S1.

### Mosquito rearing and maintenance

Newly hatched (0–3 h old) Kisumu and RSP larvae were placed individually into 60 × 12 mm glass Petri dishes containing 4 mL of permethrin at the appropriate sublethal concentration or ethanol (Fig. 1b). The larvae were fed daily with Tetramin Baby® fish food according to their age (0.04, 0.06, 0.08, 0.16, 0.32 and 0.6 mg/larva, respectively for ages 0, 1, 2, 3, 4 and 5 days or older [29]), and some developmental traits were assessed (Fig. S1). Pupae were moved to 150 mL cups (5 cm Ø × 10 cm) containing about 50 mL of deionized water and then placed in cages according to their treatment, where they emerged as adults. Adults were fed with 10% sucrose solution placed inside a 150 mL cup (5 cm Ø × 10 cm) that contained a 50 mm Ø Petri dish (to keep the mosquitoes from drowning) on the surface and two 10 × 7 cm rectangular filter paper (to allow mosquitoes to drink the solution). This cup was replaced weekly.

### Insecticide resistance

We assessed whether the resistance of adult females to permethrin-treated paper was affected by (1) prolonged larval exposure to the sublethal dose of permethrin, (2) the mosquito strain or (3) the duration of the exposure of the adults (30 or 60 min). Resistance was assessed as knock-down and mortality rates.

Males were removed from the cages within the first 24 h following their emergence to prevent any mating with the females in the experiment. For each treatment, five replicates of around 15–20 females were placed into the resting part of WHO test tubes [34]. After 2 min of acclimatization, the mosquitoes were gently blown into the exposure part of the tube containing a 0.75% permethrin-impregnated paper (WHO standard paper) for 30 or 60 min. During the exposure, we monitored the proportion of individuals that were knocked down at 5, 10, 15, 20, 25 and 30 min for all treatments and also at 45 and 60 min for the mosquitoes exposed for 60 min (Fig. 1c). After exposure, mosquitoes were gently transferred to the resting part of WHO test tubes with constant access to a 10% sucrose solution, and mortality during the 24 h after exposure was recorded.

### Choice of protected or unprotected host

We assessed whether the choice of a blood-seeking mosquito between a protected or unprotected host was affected by (1) prolonged larval exposure to a sublethal dose of permethrin, (2) the mosquito strain or (3) the mosquito’s age.

The females were reared as described above. Once the pupae had been put in cages, the females were maintained with continuous access to a 10% sucrose solution. So that the treatment of the males could have no impact on the females’ host-seeking behaviour, we removed the males from the cages within a day of emergence and replaced them with males that been reared with no exposure to the insecticide. The mosquitoes were left to mate for 5 or 15 days at a 1:1 male-to-female ratio, and we assessed the females’ host-seeking behaviour with a two-way choice apparatus [27], where mosquitoes were able to choose to blood feed either through a permethrin-treated net (Olyset Plus®) or an untreated net (Pharmavoyage® Trek). A ventilator guided the odours (at a speed of about 20 cm/s) from the two lateral cages, which contained the nets, into a central cage, into which a cup containing about 20 mosquitoes had been placed. After 2 min of acclimatization, the mosquitoes were released and given 10 min to make a choice. For each mosquito, we noted whether they left the central cage, and for the ones that did, the side they chose (with or without permethrin) and whether they took a blood meal. Finally, blood-fed mosquitoes were frozen at −20 °C for further analysis of the blood-meal size. The side of the insecticide was alternated among tests so that any side preference was avoided (Fig. 1d).

### Motivation and ability to bite through a permethrin-treated net

We assessed whether the mosquitoes’ motivation and ability to bite through a net was affected by (1) prolonged larval exposure to a sublethal dose of permethrin, (2) the mosquito strain or (3) the treatment of the net (permethrin-treated versus untreated).

The females were reared as described above, and again, only males that had not been exposed to the insecticide were allowed to mate with the females. Mating was allowed for 7 days, and on the seventh day, we let the females blood-feed for 5 min on T.G.Z.’s arm (Fig. 1e) either through a permethrin-treated net (Olyset Plus®) or through an untreated net (Pharmavoyage® Trek). We measured the proportion of mosquitoes that took a blood meal on each of the two nets, and the mosquitoes were then frozen at −20 °C for further analysis of the blood-meal size.

### Choice of egg-laying site

Finally, we assessed whether the egg-laying behaviour was affected by (1) prolonged larval exposure to a sublethal dose of permethrin or (2) the mosquito strain.

The females and males were reared as described above. A total of 7 days after emergence, the females were blood-fed on T.G.Z.’s arm for 5 min. Then, 2 days later they were individually placed into cages, given continuous access to a 10% sucrose solution, and given the possibility for 2 days to lay their eggs into a cup (5 cm Ø × 10 cm) containing about 50 mL of deionized water or into one with 50 mL of permethrin solution (Fig. 1f). The concentration of the permethrin solution was twice the sublethal dose, 0.10 mg/L for Kisumu and 1.5 mg/L for RSP. Then, the cups were collected and filtrated, and a picture was taken of the eggs. Eggs were counted using the software ImageJ v1.54 [35, 36].

### Quantification of blood-meal size

Blood-meal size was quantified as previously described [37]. We added a 5 mm stainless steel bead and 0.5 mL of Drabkin’s reagent to the standards and the samples and grounded them until disintegration with a Qiagen TissueLyser LT at a frequency of 30 Hz for 2 min. After 20 min of incubation at 25 °C, 0.5 mL of pure chloroform solution was added. We then centrifuged the samples at 4200 *g* for 5 min. The supernatant (approximately 430 µL) containing cyanmethemoglobin (HiCN) was transferred to a 2 mL reaction tube and vortexed. Then, 200 µL was placed into each of two wells of a microplate so that we could analyse two technical replicates for the reading of each mosquito and the standards. The absorbance was measured at 550 nm with a microplate spectrophotometer (SpectraMax i3x plate reader). To ensure accuracy, the standard curves were prepared using the same blood source (T.G.Z.’s blood) that was used for mosquito feeding.

### Statistical analysis

All analyses were done with the R software [38] version 4.3.0, using the packages “DHARMa” [39], “car” [40], “lme4” [41], “emmeans” [42], “multcomp” [43] and “survival” [44]. Significance was assessed with the “Anova” function of the “car” package [40]. We used a type III analysis of variance (ANOVA) in the case of a significant interaction and a type II ANOVA otherwise. When relevant, we performed post hoc multiple comparisons with the package “emmeans”, using the default Tukey adjustment.

*Determination of sublethal doses of permethrin:* The sublethal dose was analysed with a generalized linear model with a binomial distribution of errors, where the response variable was the proportion of dead larvae, and the explanatory variables were larval exposure to permethrin and the concentration.

*Insecticide resistance:* Knock-down rate was analysed with a Cox’s proportional hazard model from the survival library [44], where the response variable was age at knock-down; the explanatory variables were strain, larval exposure to permethrin and duration of adults’ exposure; and the tube in which the mosquito were exposed was a random factor. The proportional hazard ratio assumption was tested using the function cox.zph from the survival library [44]. Tubes were tested as a random factor beforehand.

Adult mortality 24 h after exposure was analysed with a generalized linear model with a binomial distribution of errors, where the response variable was the proportion of dead individuals and the explanatory variables were strain, larval exposure to permethrin and duration of adults’ exposure.

*Choice of protected or unprotected host:* Individuals (1) leaving the central cage and (2) choosing the untreated net side and (3) where individuals took a blood meal were analysed with a generalized linear model with a binomial distribution of errors, where the response variables were the proportion of individuals that (1) left the central cage, (2) went to the side with the untreated net and (3) took a blood meal, and the explanatory variables were strain, larval exposure to permethrin and the length of time that they were allowed to mate.

Blood-meal size was analysed with a linear model with a Gaussian distribution of errors, where the response variable was the individual quantity of haemoglobin in μg/mosquito and the explanatory variables were strain, larval exposure to permethrin and the length of time that they were allowed to mate.

*Motivation and ability to bite through a permethrin-treated net:* Individual motivation to take a blood meal was analysed with a generalized linear model with a binomial distribution of errors, where the response variable was the proportion of individuals that took a blood meal and the explanatory variables were strain, larval exposure to permethrin and the type of net they could bite through.

Blood-meal size was analysed with a linear model with a Gaussian distribution of errors, where the response variable was the individual quantity of haemoglobin in μg/mosquito, and the explanatory variables were strain, larval exposure to permethrin and the type of net they could bite through.

*Choice of egg-laying site:* Individuals that (1) did not lay any eggs, (2) laid eggs in both egg-layers and (3) chose the insecticide-laced egg-layer were analysed with three generalized linear models with a binomial distribution of errors, where the response variables were (1) the proportion of individuals that did not laid any eggs or, for mosquitoes that laid eggs, (2) the proportion of individuals that laid eggs in both egg-laying cups or (3) the proportion of eggs on the insecticide-laced egg-laying cup, and the explanatory variables were strain and larval exposure to permethrin.

The number of eggs was analysed with a linear model with a Gaussian distribution of errors, where the response variable was individual egg count, and the explanatory variables were strain and larval exposure to permethrin.

## Results

### Insecticide resistance

We exposed adult mosquitoes (690 Kisumu mosquitoes and 507 RSP mosquitoes) to 0.75% permethrin-impregnated paper in WHO test tubes for 30 or 60 min, and assessed whether they were knocked down during the exposure and whether they died within 24 h after the exposure (Fig. 1c).

Kisumu mosquitoes were significantly more sensitive to permethrin than RSP mosquitoes and were knocked down faster (median knock-down time: 15 min versus 60 min, *χ*^2^ = 531.24, *df* = 1; *p* < 0.001). Of the Kisumu mosquitoes 94% were knocked down by 30 min and 100% by 60 min, whereas of RSP mosquitoes 11% were knocked down by 30 min and 80% by 60 min (interaction strain × duration: *χ*^2^ = 20.64, *df *= 1; *p* < 0.001).

Of the Kisumu mosquitoes, 96% died within 24 h of exposure, whereas 53% of RSP mosquitoes did (*χ*^2^ = 180.83, *df* = 1; *p* < 0.001). Mortality increased with exposure time in RSP mosquitoes (31% at 30 min versus 75% at 60 min), but remained consistently high in Kisumu mosquitoes (interaction strain × duration of exposure: *χ*^2^ = 20.65, *df* = 1; *p* < 0.001).

**Fig. 2:**
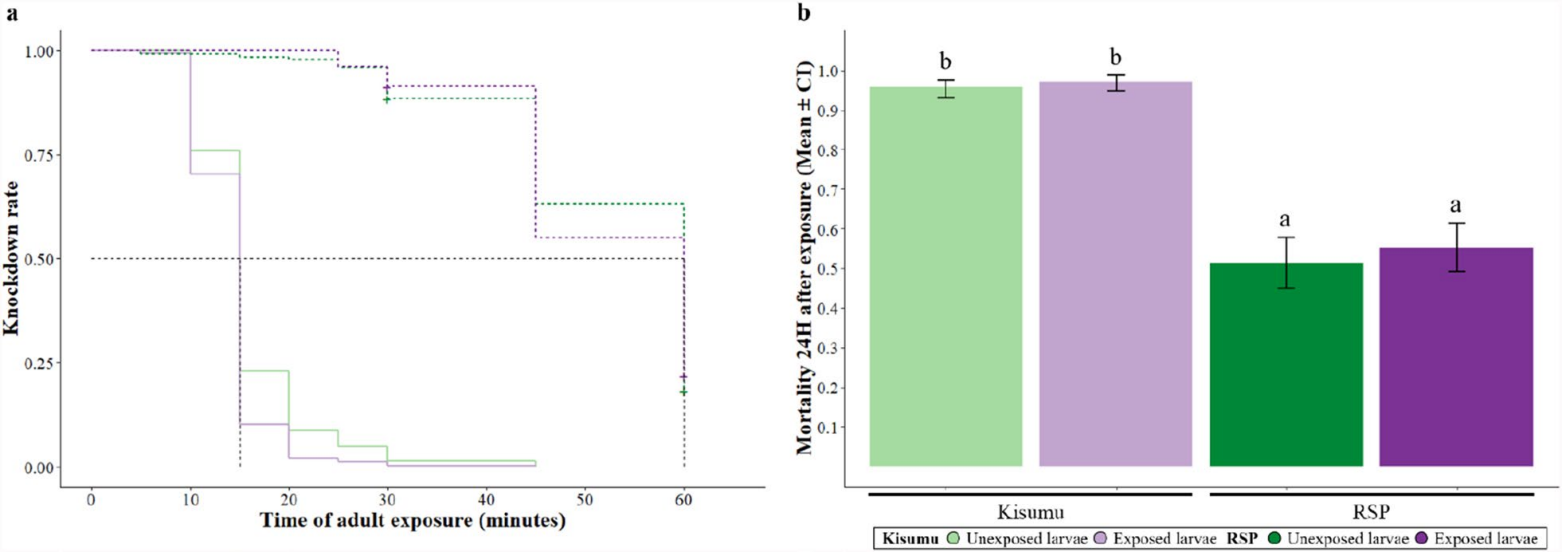
**Insecticide resistance.** The proportion of individuals **a** knocked down or **b** dead 24 h after their adult exposure for each strain (dotted lines represent RSP individuals, while solid lines correspond to Kisumu) and larval exposure treatment. Error bars show the 95% confidence intervals of the means. Letters indicate statistically significant differences from multiple comparisons. For details on the sample size, see Supplementary Table S1.2

Interestingly, larval exposure to permethrin did not significantly influence knock-down times (*χ*^2^ = 0.27, *df* = 1; *p* = 0.605, Fig. 2a) or mortality (78% in both exposed and unexposed groups, *χ*^2^ = 2.34, *df* = 1; *p* = 0.125; Fig. 2b). For further details on the statistical analysis, see Supplementary Table S1.1.

### Choice of protected or unprotected host

We tested 687 mosquitoes in the choice assay (Fig. 1d).

**Fig. 3:**
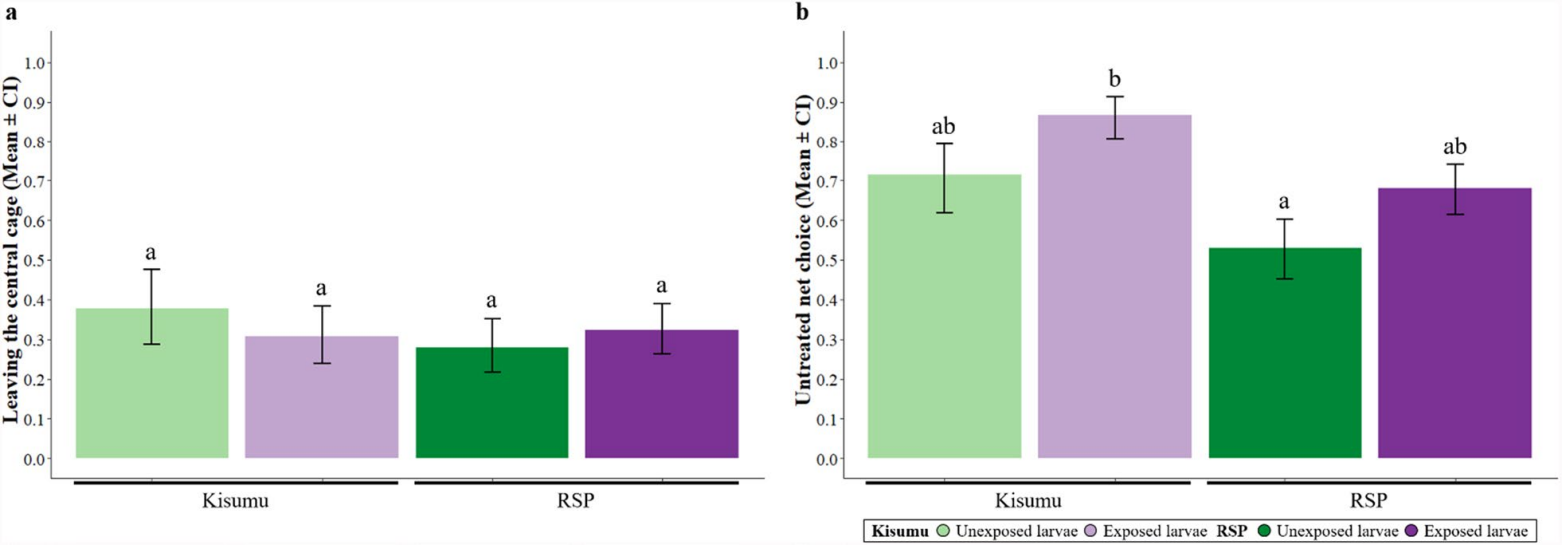
**Choice of protected or unprotected host. **The proportion of individuals that **a** left the central cage and **b** chose the untreated net side for each strain and larval exposure. Error bars show the 95% confidence intervals of the means. Letters indicate statistically significant differences from multiple comparisons. For details on the sample size, see Supplementary Table S2.2

Of the mosquitoes 32% left the central cage independently of their strain (*χ*^2^ = 1.41, *df* = 1; *p* = 0.234), of their exposure to permethrin (*χ*^2^ = 0.97, *df* = 1; *p* = 0.322) or of the interaction between the two (interaction strain × larval exposure: *χ*^2^ = 3.58, *df* = 1; *p* = 0.058; Fig. 3a).

Among the 218 mosquitoes that left the central cage, Kisumu mosquitoes were significantly more likely to choose the untreated net than RSP mosquitoes (80% versus 61%, *χ*^2^ = 4.22, *df* = 1; *p* = 0.039). Additionally, mosquitoes exposed to permethrin as larvae had a stronger preference for the untreated net than unexposed ones (76% versus 61%, *χ*^2^ = 6.08, *df* = 1; *p* = 0.013). This difference was consistent across the two strains (86% for exposed versus 71% for unexposed for Kisumu, 68% for exposed versus 52% for unexposed RSP; interaction strain × exposure: *χ*^2^ = 0.23, *df* = 1; *p* = 0.626; Fig. 3b). For further details on the statistical analysis, see Supplementary Table S2.1.

### Motivation and ability to bite through a permethrin-treated net

We assessed the blood-feeding behaviour in a total of 582 females (Fig. 1e).

**Fig. 4:**
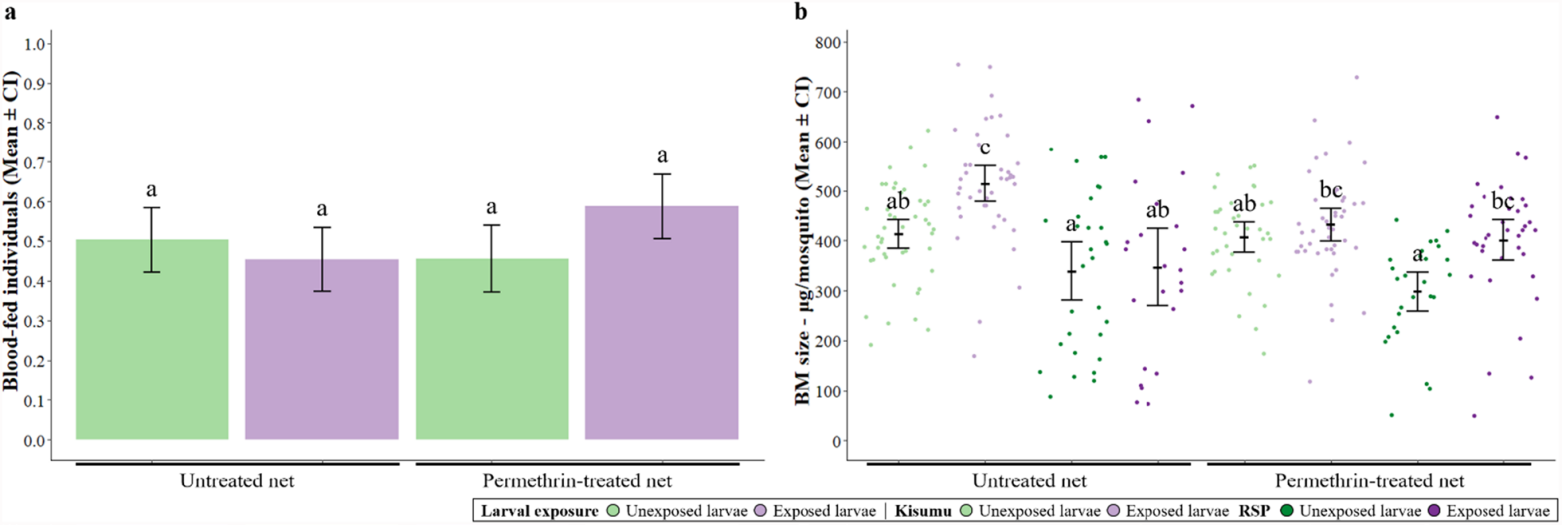
**Motivation and ability to bite through a permethrin-treated net.** **a** The proportion of individuals that took a blood meal through a permethrin-treated or untreated net for each larval exposure treatment, independently of the strain. **b** Individual blood-meal size (i.e. quantity of haemoglobin) for each larval exposure treatment and strain. Error bars show the 95% confidence intervals of the means. Letters indicate statistically significant differences from multiple comparisons. For details on the sample size, see Supplementary Table S3.2

In all, 57% of Kisumu mosquitoes and 42% of RSP mosquitoes took a blood meal (*χ*^2^ = 13.84, *df* = 1; *p* < 0.001). If given access to a treated net RSP mosquitoes were more likely to bite than if they were given access to an untreated one (50% through treated net versus 35% through untreated net), whereas the type of net had only a small impact on Kisumu mosquitoes (54% treated versus 60% untreated; interaction strain × net: *χ*^2^ = 5.79, *df* = 1; *p* = 0.016). Larval exposure did not influence overall feeding rates (*χ*^2^ = 0.92, *df* = 1; *p* = 0.335), but exposed mosquitoes were more likely to feed through a treated net than unexposed ones (58% treated versus 45% untreated for exposed mosquitoes, 45% treated versus 50% untreated for unexposed; interaction exposure × net: *χ*^2^ = 4.74, *df* = 1; *p* = 0.029; Fig. 4a).

Among those that fed, Kisumu mosquitoes ingested more blood than RSP mosquitoes (467.43 μg versus 357.85 μg, F_1,283_ = 3.54, *df* = 1; *p* = 0.060). Mosquitoes exposed as larvae also took larger blood meals than unexposed ones (467.36 μg versus 373.02 μg, F_1,283_ = 17.93, *df* = 1; *p* < 0.001), in particular in Kisumu mosquitoes (interaction exposure × strain: F_1,283_ = 5.99, *df* = 1; *p* = 0.014). While blood-meal size was generally unaffected by net type (F_1,283_ = 0.03, *df* = 1, *p* = 0.852), it depended on strain and larval exposure (interaction exposure × strain × net: F_1,283_ = 6.04, *df* = 1, *p* = 0.014, Fig. 4b). For further details on the statistical analysis, see Supplementary Table S3.1.

### Choice of egg-laying site

After blood-feeding, 353 females were tested for their egg-laying behaviour (Fig. 1f).

**Fig. 5:**
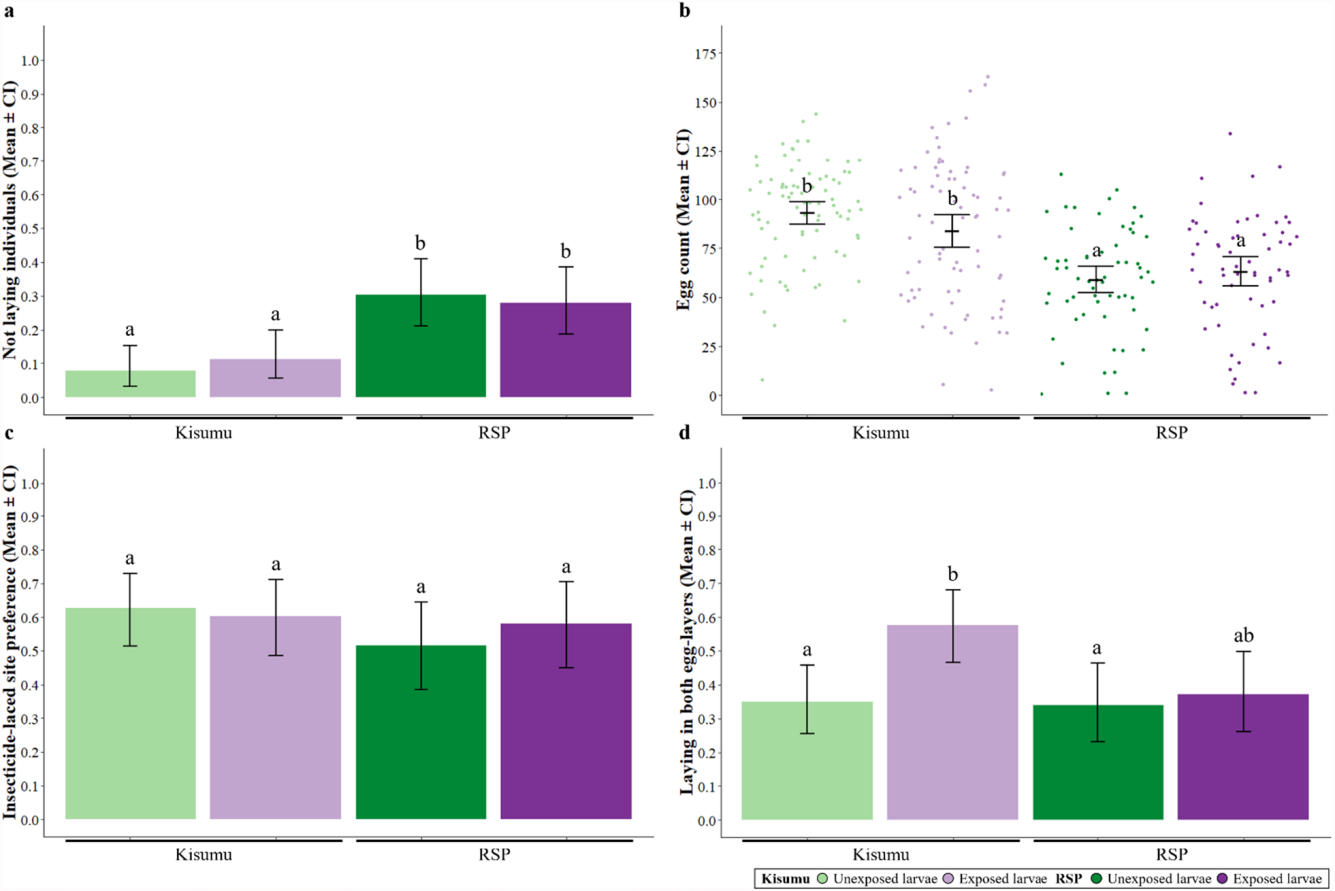
**Choice of egg-laying site. **The proportion of Kisumu and RSP individuals, exposed or unexposed to permethrin during the larval stage that **a** did not lay eggs or **d** laid in both egg-layers, as well as **b** the total egg count for each individual and **c** the proportion of eggs laid in the insecticide-laced site. Error bars show the 95% confidence intervals of the means. Letters indicate statistically significant differences from multiple comparisons. For details on the sample size, see Supplementary Table S4.2

Of the females, 19% did not lay eggs. This was significantly higher in RSP mosquitoes (29%) than in Kisumu mosquitoes (9%) (*χ*^2^ = 22.58, *df* = 1; *p* < 0.001), but was unaffected by larval exposure (*χ*^2^ = 0.02, *df* = 1; *p* = 0.882; Fig. 5a). Those that laid eggs laid an average of 76 eggs, but RSP mosquitoes laid fewer eggs than Kisumu mosquitoes (62 versus 86, F_1,281_ = 58.09, *df* = 1; *p* < 0.001) and larval exposure did not influence egg-laying behaviour (F_1,281_ = 0.93, *df* = 1; *p* = 0.333; Fig. 5b).

Of the laid eggs, 57% were deposited in the insecticide-laced water, with no significant effects of strain (*χ*^2^ = 1.28, *df* = 1; *p* = 257) or larval exposure (*χ*^2^ = 0.06, *df* = 1; *p* = 0.882; Fig. 5c). However, 41% of mosquitoes laid in both cups (skip oviposition). This behaviour was more common in exposed mosquitoes (48%) than unexposed ones (34%) (*χ*^2^ = 6.05, *df* = 1; *p* = 0.013), particularly for Kisumu mosquitoes (*χ*^2^ = 3.39, *df* = 1; *p* = 0.065, Fig. 5d). For further details on the statistical analysis, see Supplementary Table S4.1.

## Discussion

Larval exposure to sublethal doses of insecticides significantly affected the physiology and behaviour of adult mosquitoes. Given that permethrin residues are commonly found in surface waters at concentrations ranging from 0.003 µg/L to 0.80 µg/L [3, 4], the sublethal doses used in our study fall well within this environmental range. Notably, while unexposed mosquitoes avoided biting through a permethrin-treated net, those exposed during the larval stage preferred seeking hosts behind untreated nets when given a choice between treated and untreated protection (Fig. 3). However, when they were given no choice, exposed mosquitoes were more likely to take a blood meal through a treated net (Fig. 4). Exposed individuals exhibited increased skip oviposition behaviour (i.e. laying eggs in both available sites; Fig. 5). However, resistance, assessed as knock-down and mortality rates, was not affected by larval exposure (Fig. 2).

In contrast to other larval stressors such as nutrition [29] and parasitism [27], larval exposure to permethrin did not affect insecticide resistance of the susceptible Kisumu or the resistant RSP mosquitoes (Fig. 2). Although larval exposure influences oxidative homeostasis [45], which is crucial for resistance, our results align with previous studies indicating no impact on mortality from subsequent insecticide exposure [26].

When given a choice between a host protected by a treated or an untreated net (Fig. 3b), Kisumu mosquitoes avoided the treated one, highlighting their inherent sensitivity to insecticides [46]. Conversely, RSP mosquitoes did not show this avoidance, likely due to the *kdr* mutation, which reduces the efficacy of insecticide-targeting voltage-gated sodium channels and alters the neural circuits, and therefore affects the mosquitoes’ sensitivity and the repellence of environmental cues [22, 23]. The difference between Kisumu and RSP corroborates several studies showing that resistant mosquitoes typically are repelled less than sensitive ones [47–49]. Sublethal larval exposure to permethrin increased the repellency of the insecticide to the adults of both strains. A mechanism for this response would be that pyrethroid exposure affects the neural system in insects [50], altering their response to olfactory cues [19, 20]. Our results thus suggest that this effect is long term, lasting from the exposure in larvae to the adult stage. Larval exposure thus sensitizes mosquitoes in a way that enhances their avoidance behaviour (i.e. excito-repellency [51, 52]) as adults, even if this behaviour is normally suppressed by a resistant mutation.

When allowed to bite either through an untreated or a treated net, but not given a choice between the two (Fig. 4), Kisumu mosquitoes bit through the two nets with a similar likelihood. In contrast, RSP mosquitoes were more likely to bite through a treated net, corroborating several studies [7, 45, 53–55]. Furthermore, unexposed individuals bit through the two nets with a similar likelihood, but exposed individuals were more likely to bite through a permethrin-treated net than through an untreated one. While this appears to contradict our idea of sensitization, larval exposure is also a stress factor that might compel mosquitoes to prioritize fecundity, thus enhancing the motivation to bite. This behaviour aligns with the natural preferences of each strain, with Kisumu mosquitoes favouring untreated nets and RSP mosquitoes favouring treated nets (Fig. 4b). Whatever the mechanism, our results indicate a complex interplay of stress responses, strain-specific adaptations and potential changes in sensory perception or behavioural conditioning due to larval insecticide exposure.

As in many studies of *Anopheles* mosquitoes [56–58], the resistant RSP mosquitoes laid fewer eggs than the sensitive Kisumu mosquitoes (Fig. 5a, b). This suggests a cost of resistance, paid by re-allocating resources away from fecundity and towards resistance. In contrast to our prediction, larval exposure did not make females avoid the insecticide-laced site during egg-laying (Fig. 5c). Rather, it increased the proportion of individuals that distributed their eggs over both egg-laying cups (Fig. 5d), suggesting that larval exposure increases the likelihood of using skip oviposition, which is defined as visiting (and laying into) several egg-laying sites [59]. While this is common behaviour in *Aedes* mosquitoes [60–62], it is rarely reported in *Anopheles* [63–65], which tend to lay all eggs in a single site. A possible reason is that larval exposure to permethrin damages the odour sensors, preventing mosquitoes from distinguishing between suitable and unsuitable laying sites [19, 20]. Alternatively, it could indicate a bet-hedging strategy, where exposed females distribute their eggs across multiple sites to increase the chances of some offspring surviving, despite the risk of some being laid in suboptimal locations. These behavioural changes, combined with the observed effects on blood-feeding motivation and host-seeking, challenge studies in which pyrethroids appear to remain repellent for resistant mosquitoes [66] and reduce the blood-meal size of mosquitoes facing treated nets [49], and suggest that sublethal insecticide exposure may have broader implications for mosquito population dynamics and vector control strategies. Further research should explore the physiological mechanisms underlying these effects and assess their impact in field conditions, where mosquitoes encounter multiple environmental stressors.

Finally, we acknowledge that in addition to the insecticide resistance status, other genetic differences between the RSP and Kisumu strains could have contributed to the observed phenotypic variation. While resistance-linked traits were a primary focus of this study, we cannot exclude the influence of broader genetic background effects.

## Conclusions

Our study demonstrates that sublethal exposure to insecticides during larval development has long-term effects on adult mosquito behaviour, particularly in their sensitivity to insecticides, blood-feeding motivation and oviposition strategies. By increasing the preference of resistant mosquitoes for insecticide-treated nets and their blood-meal size, sublethal exposure may exacerbate disease transmission dynamics and reduce the effectiveness of insecticide-based vector control strategies. These findings highlight the unintended consequences of widespread insecticide use and reinforce the need for alternative, non-insecticidal control measures to mitigate the risks associated with insecticide resistance and environmental contamination.

## Supplementary Information

Supplementary material 1.

Supplementary material 2.

Supplementary material 3.

Supplementary material 4.

Supplementary material 5.

Supplementary material 6.

## Data Availability

All data generated or analysed during this study are included as Supplementary Information files.

## References

[1] Pluess B, Tanser FC, Lengeler C, Sharp BL. Indoor residual spraying for preventing malaria. Cochrane Database of Systematic Reviews. 2010;2010. Available from: https://pubmed.ncbi.nlm.nih.gov/20393950/10.1002/14651858.CD006657.pub2 PMC6532743 20393950

[2] World Health Organization. Managing pesticides in agriculture and public health: a compendium of FAO and WHO guidelines and other resources. Managing pesticides in agriculture and public health. FAO; 2021 [cited 2024 Apr 18]. https://www.who.int/publications/i/item/9789240022478

[3] Thapinta A, Hudak PF. Pesticide use and residual occurrence in Thailand. Environ Monit Assess. 2000;60:103–14.

[4] Stehle S, Bub S, Schulz R. Compilation and analysis of global surface water concentrations for individual insecticide compounds. Sci Total Environ. 2018;639:516–25. 29800845 10.1016/j.scitotenv.2018.05.158

[5] Tiryaki O, Temur C. The Fate of Pesticide in the Environment. Journal of Biological and Environmental Sciences. 2010;4:29–38.

[6] Bantz A, Camon J, Froger JA, Goven D, Raymond V. Exposure to sublethal doses of insecticide and their effects on insects at cellular and physiological levels. Curr Opin Insect Sci. Elsevier Inc.; 2018. p. 73–8. 10.1016/j.cois.2018.09.008 30553488

[7] Andreazza F, Oliveira EE, Martins GF. Implications of Sublethal Insecticide Exposure and the Development of Resistance on Mosquito Physiology, Behavior, and Pathogen Transmission. Insects 2021, Vol 12, Page 917. 2021;12:917. Available from: https://www.mdpi.com/2075-4450/12/10/917/htm. 10.3390/insects12100917 PMC8539869 34680686

[8] Mulatier M, Ahoua Alou LP, Chandre F, Pennetier C, Dormont L, Cohuet A. Effect of DEET-multiple exposures on behavior and life history traits in the malaria mosquito Anopheles gambiae (s.s.). Parasit Vectors. 2018;11:1–10. 10.1186/s13071-018-3024-0. 10.1186/s13071-018-3024-0 PMC6060454 30045761

[9] Müller P, Chouaïbou M, Pignatelli P, Etang J, Walker ED, Donnelly MJ, et al. Pyrethroid tolerance is associated with elevated expression of antioxidants and agricultural practice in *Anopheles arabiensis* sampled from an area of cotton fields in Northern Cameroon. Mol Ecol. 2008;17:1145–55. 18179425 10.1111/j.1365-294X.2007.03617.x

[10] Belzunces LP, Tchamitchian S, Brunet JL. Neural effects of insecticides in the honey bee. Apidologie 2012 43:3. 2012;43:348–70. Available from: https://link.springer.com/article/10.1007/s13592-012-0134-0.

[11] Cohnstaedt LW, Allan SA. Effects of sublethal pyrethroid exposure on the host-seeking behavior of female mosquitoes. J Vector Ecol. 2011;36:395–403. 22129411 10.1111/j.1948-7134.2011.00180.x

[12] Diop MM, Chandre F, Rossignol M, Porciani A, Chateau M, Moiroux N, et al. Sub-lethal insecticide exposure affects host biting efficiency of Kdr-resistant Anopheles gambiae. Peer Community Journal. 2021;1. http://www.centre-mersenne.org/

[13] Yadav P, Barde P, Gokhale M, Vipat V, Mishra A, Pal J, et al. Effect of temperature and insecticide stresses on *Aedes aegypti* larvae and their influence on the susceptibility of mosquitoes to dengue-2 virus. Southeast Asian J Trop Med Public Health. 2005;36:1139. 16438138

[14] Muturi EJ, Alto BW. Larval environmental temperature and insecticide exposure alter *Aedes aegypti* competence for arboviruses. Vector-Borne Zoonotic Dis. 2011;11:1157–63. 21453010 10.1089/vbz.2010.0209

[15] Vantaux A, Ouattarra I, Lefèvre T, Dabiré KR. Effects of larvicidal and larval nutritional stresses on Anopheles gambiae development, survival and competence for Plasmodium falciparum. Parasit Vectors. 2016;9:1–11.10.1186/s13071-016-1514-5. 10.1186/s13071-016-1514-5 PMC4842262 27107591

[16] Khalil H, Rifaat M, Sadek S, Gad A. Effect of the sublethal concentrations of the insecticides DDT, Abate and Sevin on the filaria cycle in *Culex pipiens* molestus. J Egypt Public Health Assoc. 1975;50:309–18.

[17] Hauser G, Thiévent K, Koella JC. Consequences of larval competition and exposure to permethrin for the development of the rodent malaria *Plasmodium berghei* in the mosquito *Anopheles gambiae*. Parasit Vectors. 2020;13:1–11. 32106886 10.1186/s13071-020-3983-9 PMC7045583

[18] Parkinson RH, Little JM, Gray JR. A sublethal dose of a neonicotinoid insecticide disrupts visual processing and collision avoidance behaviour in Locusta migratoria. Sci Rep. 2017;7:1–13. 10.1038/s41598-017-01039-1. 10.1038/s41598-017-01039-1 PMC5430526 28428563

[19] Tricoire-Leignel H, Thany SH, Gadenne C, Anton S. Pest insect olfaction in an insecticide-contaminated environment: Info-disruption or hormesis effect. Front Physiol. 2012;3 MAR:23335. 10.3389/fphys.2012.00058. 10.3389/fphys.2012.00058 PMC3307139 22457653

[20] Williamson SM, Wright GA. Exposure to multiple cholinergic pesticides impairs olfactory learning and memory in honeybees. Journal of Experimental Biology. 2013;216:1799–807. 10.1242/jeb.083931 PMC3641805 23393272

[21] Afify A, Galizia CG. Chemosensory cues for mosquito oviposition site selection. J Med Entomol. 2015;52:120–30. 26336295 10.1093/jme/tju024

[22] Bentley MD, Day JF. Chemical ecology and behavioral aspects of mosquito oviposition further annual reviews. Ann Rev Entomol. 1989. www.annualreviews.org 10.1146/annurev.en.34.010189.002153 2564759

[23] Schoelitsz B, Mwingira V, Mboera LEG, Beijleveld H, Koenraadt CJM, Spitzen J, et al. Chemical mediation of oviposition by *Anopheles *mosquitoes: a push-pull system driven by volatiles associated with larval stages. J Chem Ecol. 2020;46:397–409. 32240482 10.1007/s10886-020-01175-5 PMC7205850

[24] Margus A, Piiroinen S, Lehmann P, Tikka S, Karvanen J, Lindström L. Sublethal Pyrethroid Insecticide Exposure Carries Positive Fitness Effects Over Generations in a Pest Insect. Sci Rep. 2019;9:1–10. 10.1038/s41598-019-47473-1. 10.1038/s41598-019-47473-1 PMC6683203 31383885

[25] Iftikhar A, Hafeez F, Aziz MA, Hashim M, Naeem A, Yousaf HK, et al. Assessment of sublethal and transgenerational effects of spirotetramat, on population growth of cabbage aphid, Brevicoryne brassicae L. (Hemiptera: Aphididae). Front Physiol. 2022;13:1014190.10.3389/fphys.2022.1014190. 10.3389/fphys.2022.1014190 PMC9791945 36579021

[26] Glunt KD, Thomas MB, Read AF. The effects of age, exposure history and malaria infection on the susceptibility of *Anopheles* mosquitoes to low concentrations of pyrethroid. PLoS One. 2011;6:e24968. 21966392 10.1371/journal.pone.0024968 PMC3178580

[27] Thiévent K, Hofer L, Rapp E, Tambwe MM, Moore S, Koella JC. Malaria infection in mosquitoes decreases the personal protection offered by permethrin-treated bednets. Parasit Vectors. 2018;11:284. 29728155 10.1186/s13071-018-2846-0 PMC5936035

[28] Grossman MK, Uc-Puc V, Flores AE, Manrique-Saide PC, Vazquez-Prokopec GM. Larval density mediates knockdown resistance to pyrethroid insecticides in adult *Aedes aegypti*. Parasit Vectors. 2018;11. 10.1186/s13071-018-2865-x PMC5934844 29724237

[29] Kulma K, Saddler A, Koella JC. Effects of age and larval nutrition on phenotypic expression of insecticide-resistance in *Anopheles* mosquitoes. PLoS ONE. 2013;8:e58322. 23484017 10.1371/journal.pone.0058322 PMC3590143

[30] Vulule JM, Beach RF, Atieli FK, Roberts JM, Mount DL, Mwangi RW. Reduced susceptibility of *Anopheles gambiae* to permethrin associated with the use of permethrin-impregnated bednets and curtains in Kenya. Med Vet Entomol. 1994;8:71–5. 8161849 10.1111/j.1365-2915.1994.tb00389.x

[31] Vulule JM, Beach RF, Atieli FK, Mount DL, Roberts JM, Mwangi RW. Long-term use of permethrin-impregnated nets does not increase Anopheles gambiae permethrin tolerance. Med Vet Entomol. 1996;10:71–9. 10.1111/j.1365-2915.1996.tb00084.x. 10.1111/j.1365-2915.1996.tb00084.x 8834745

[32] Vulule JM, Beach RF, Atieli FK, McAllister JC, Brogdon WG, Roberts JM, et al. Elevated oxidase and esterase levels associated with permethrin tolerance in *Anopheles gambiae* from Kenyan villages using permethrin-impregnated nets. Med Vet Entomol. 1999;13:239–44. 10514048 10.1046/j.1365-2915.1999.00177.x

[33] Sharom MS, Solomon KR. Adsorption and desorption of permethrin and other pesticides on glass and plastic materials used in bioassay procedures. Can J Fisheries Aquatic Sci. 1981;38. www.nrcresearchpress.com

[34] World Health Organization. Test procedures for insecticide resistance monitoring in malaria vector mosquitoes. Geneva: World Health Organization; 2016.

[35] Abràmoff MD, Magalhães PJ, Ram SJ. Image processing with ImageJ. Biophotonics international. 2004;11:36–42.

[36] Zeferino TG, Koella JC. Host-specific effects of a generalist parasite of mosquitoes. Sci Rep. 2024;14:18365. https://www.nature.com/articles/s41598-024-69475-4 10.1038/s41598-024-69475-4 PMC11306583 39112600

[37] Briegel H, Lea AO, Klowden MJ. Hemoglobinometry as a method for measuring blood meal sizes of mosquitoes (Diptera: Culicidae). J Med Entomol. 1979;15:235–8. https://academic.oup.com/jme/article/15/3/235/2219507 10.1093/jmedent/15.5-6.514 44528

[38] Team RC. R: A language and environment for statistical computing. Vienna, Austria: R Foundation for Statistical Computing; 2017.

[39] Hartig F. DHARMa: Residual diagnostics for hierarchical (multi-level/mixed) regression models. 2022. https://CRAN.R-project.org/package=DHARMa

[40] Fox J, Weisberg S. Using car and effects functions in other functions. Published online. 2020;1–5.

[41] Bates D, Mächler M, Bolker B, Walker S. Fitting linear mixed-effects models using lme4. 2014; http://arxiv.org/abs/1406.5823

[42] Lenth R V. Emmeans: estimated marginal means, aka least-squares means. 2023. https://CRAN.R-project.org/package=emmeans

[43] Hothorn T, Bretz F, Westfall P. Simultaneous inference in general parametric models. Biom J. 2008;50:346–63. 18481363 10.1002/bimj.200810425

[44] Therneau T, et al. A package for survival analysis in S. R Package Version. 2015;2:1.

[45] Carrasco D, Lefèvre T, Moiroux N, Pennetier C, Chandre F, Cohuet A. Behavioural adaptations of mosquito vectors to insecticide control. Curr Opin Insect Sci. 2019;34:48–54. Available from: https://www.sciencedirect.com/science/article/pii/S2214574518301469 10.1016/j.cois.2019.03.005 31247417

[46] Sfara V, Mougabure-Cueto G, Zerba EN, Alzogaray RA. Adaptation of the repellency response to DEET in *Rhodnius prolixus*. J Insect Physiol. 2011;57:1431–6. 21801727 10.1016/j.jinsphys.2011.07.009

[47] Fathy Khater H, M. Selim A, A. Abouelella G, A. Abouelella N, Murugan K, P. Vaz N, et al. Commercial Mosquito Repellents and Their Safety Concerns. Malaria. IntechOpen; 2019. Available from: 10.5772/intechopen.87436.

[48] Wagman JM, Achee NL, Grieco JP. Insensitivity to the spatial repellent action of transfluthrin in *Aedes aegypti*: a heritable trait associated with decreased insecticide susceptibility. PLoS Negl Trop Dis. 2015;9:3. 10.1371/journal.pntd.0003726 PMC4400042 25879206

[49] Barreaux P, Ranson H, Foster GM, McCall PJ. Pyrethroid-treated bed nets impair blood feeding performance in insecticide resistant mosquitoes. Sci Rep. 2023;13:1–11. 10.1038/s41598-023-35958-z. 10.1038/s41598-023-35958-z PMC10284836 37344580

[50] Lucas P, Renou M. Electrophysiological study of the effects of deltamethrin, bioresmethrin, and DDT on the activity of pheromone receptor neurones in two moth species. Pestic Biochem Physiol. 1992;43:103–15. 10.1016/0048-3575(92)90024-T.

[51] Contreras-Perera YJ, Martin-Park A, Puerta-Guardo H, Che-Mendoza A, Pérez-Carrillo S, Rodríguez-Sánchez IP, et al. Mosquito Excito-Repellency: Effects on Behavior and the Development of Insecticide Resistance. Mosquito Research - Recent Advances in Pathogen Interactions, Immunity, and Vector Control Strategies. 2022; Available from: https://www.intechopen.com/chapters/82438

[52] Sukkanon C, Nararak J, Bangs MJ, Hii J, Chareonviriyaphap T. Behavioral responses to transfluthrin by Aedes aegypti, Anopheles minimus, Anopheles harrisoni, and Anopheles dirus (Diptera: Culicidae). PLoS One. 2020;15:e0237353. 10.1371/journal.pone.0237353. 10.1371/journal.pone.0237353 PMC7423148 32785255

[53] Diop MM, Moiroux N, Chandre F, Martin-Herrou H, Milesi P, Boussari O, et al. Behavioral Cost & Overdominance in Anopheles gambiae. PLoS One. 2015;10:e0121755. 10.1371/journal.pone.0121755. 10.1371/journal.pone.0121755 PMC4382092 25831058

[54] Porciani A, Diop M, Moiroux N, Kadoke-Lambi T, Cohuet A, Chandre F, et al. Influence of pyrethroïd-treated bed net on host seeking behavior of Anopheles gambiae s.s. carrying the kdr allele. PLoS One. 2017;12:e0164518. 10.1371/journal.pone.0164518. 10.1371/journal.pone.0164518 PMC5536278 28759566

[55] Vais H, Williamson MS, Goodson SJ, Devonshire AL, Warmke JW, Usherwood PNR, et al. Activation of Drosophila Sodium Channels Promotes Modification by DeltamethrinReductions in Affinity Caused by Knock-down Resistance Mutations. Journal of General Physiology. 2000;115:305–18. http://www.jgp.org/cgi/content/full/115/3/305 10.1085/jgp.115.3.305 PMC2217214 10694259

[56] Osoro JK, Machani MG, Ochomo E, Wanjala C, Omukunda E, Githeko AK, et al. Insecticide resistant Anopheles gambiae have enhanced longevity but reduced reproductive fitness and a longer first gonotrophic cycle. Sci Rep. 2022;12:1–7. 10.1186/s13071-018-3065-4. 10.1038/s41598-022-12753-w PMC9126871 35606505

[57] Sy FA, Faye O, Diallo M, Dia I. Effects of insecticide resistance on the reproductive potential of two sub-strains of the malaria vector *Anopheles coluzzii*. J Vector Borne Dis. 2019;56:207–11. http://journals.lww.com/jvbd 10.4103/0972-9062.289401 32655069

[58] Nkahe DL, Kopya E, Djiappi-Tchamen B, Toussile W, Sonhafouo-Chiana N, Kekeunou S, et al. Fitness cost of insecticide resistance on the life-traits of a *Anopheles coluzzii* population from the city of Yaoundé. Cameroon Wellcome Open Res. 2020;5:171. 33029560 10.12688/wellcomeopenres.16039.1 PMC7525343

[59] Mogi M, Mokry J. Distribution of *Wyeomyia smithii* (Diptera, Culicidae) eggs in pitcher plants in Newfoundland. Canada Trop Med Nagasaki Univ. 1980;22:1–12.

[60] Colton YM, Chadee DD, Severson DW. Natural skip oviposition of the mosquito *Aedes aegypti* indicated by codominant genetic markers. Med Vet Entomol. 2003;17:195–204. 12823837 10.1046/j.1365-2915.2003.00424.x

[61] Corbet PS, Chadee DD. An improved method for detecting substrate preferences shown by mosquitoes that exhibit ‘skip oviposition.’ Physiol Entomol. 1993;18:114–8.

[62] Bibbs CS, Hahn DA, Kaufman PE, Xue R De. Sublethal effects of a vapour-active pyrethroid, transfluthrin, on Aedes aegypti and Ae. albopictus (Diptera: Culicidae) fecundity and oviposition behaviour. Parasit Vectors. 2018;11:1–9. 10.1186/s13071-018-3065-4. 10.1186/s13071-018-3065-4 PMC6114540 30157907

[63] Hong C, Ulrike F, Guiyan Y. Oviposition behavior of female *Anopheles gambiae* in western Kenya inferred from microsatellitemarkers. Am Soc Trop Med Hygiene. 2006;75:246–50. 16896126

[64] Ogbunugafor CB, Sumba L. Behavioral evidence for the existence of a region-specific oviposition cue in *Anopheles gambiae* s.s. J Vector Ecol. 2008;33:321–4. 19263852 10.3376/1081-1710-33.2.321

[65] Okal MN, Lindh JM, Torr SJ, Masinde E, Orindi B, Lindsay SW, et al. Analysing the oviposition behaviour of malaria mosquitoes: Design considerations for improving two-choice egg count experiments. Malar J. 2015;14:1–17. 10.1186/s12936-015-0768-2. 10.1186/s12936-015-0768-2 PMC4474426 26088669

[66] Bowman NM, Akialis K, Cave G, Barrera R, Apperson CS, Meshnick SR. Pyrethroid insecticides maintain repellent effect on knock-down resistant populations of *Aedes aegypti* mosquitoes. PLoS One. 2018;13:e0196410. 29763445 10.1371/journal.pone.0196410 PMC5953453

